# Occurrence and Predictors of Bacterial Respiratory Tract Infections and Antimicrobial Resistance Among Isolates From Dogs Presented With Lower Respiratory Tract Infections at a Referral Veterinary Hospital in South Africa

**DOI:** 10.3389/fvets.2020.00304

**Published:** 2020-06-02

**Authors:** Daniel Nenene Qekwana, Vinny Naidoo, James Wabwire Oguttu, Agricola Odoi

**Affiliations:** ^1^Section Veterinary Public Health, Department of Paraclinical Sciences, Faculty of Veterinary Science, University of Pretoria, Pretoria, South Africa; ^2^Faculty of Veterinary Science, University of Pretoria Biomedical Research Centre, Pretoria, South Africa; ^3^Department of Agriculture and Animal Health, College of Agriculture and Environmental Sciences, University of South Africa, Johannesburg, South Africa; ^4^Department of Biomedical and Diagnostic Sciences, College of Veterinary Medicine, University of Tennessee, Knoxville, Knoxville, TN, United States

**Keywords:** respiratory tract infections, dog, canine, antimicrobial resistance, multidrug resistance, logistic regression, Generalized Estimating Equations, South Africa

## Abstract

**Background:** Respiratory tract infections (RTIs) associated with *Pasteurella multocida, Bordetella bronchiseptica*, Streptococci, Staphylococci, and *Pseudomonas* species have been reported in dogs. The objective of this study was to investigate the occurrence and predictors of bacterial RTIs and antimicrobial resistance among samples from dogs with lower RTIs at a referral veterinary teaching hospital in South Africa.

**Methods:** Records of 157 dogs with lower RTIs presented to the veterinary teaching hospital between 2007 and 2013 were included in the study. Crude and factor-specific proportions of RTIs and antimicrobial resistance by breed, season, year, sex, age category, and specimen type were computed. Chi-square or Fisher's exact tests were used to compare proportions of RTIs and antimicrobial resistant isolates across categorical variables. Associations between breed, season, year, sex, age, specimen, and odds of RTIs or multidrug resistance were assessed using Generalized Estimating Equations.

**Results:** There was only one sample per clinical case and bacterial RTIs were observed in 53.5% of the samples tested. *Pasteurella* species (23.5%) were more common than other species. Almost all (99.5%) isolates were resistant to at least one antimicrobial, while 64.7% were multidrug resistant (MDR). Additionally, 17.0% and 3.3% showed evidence of extensive drug resistance (XDR) and pan-drug resistance (PDR), respectively. The majority of MDR isolates were resistant to penicillin-G (90.9%), lincomycin (100%), tylosine (75.8%), lincospectin (73.7%), ampicillin (72.5%), and kanamycin (68.4%). None of the investigated predictors had significant association with RTIs or antimicrobial resistance.

**Conclusion:** *Pasturella* species were the most common causes of RTIs. The high levels of MDR and the presence of both XDR and PDR isolates raise the question of the effectiveness of the current antimicrobial therapy used in patients with RTIs in referral hospitals. Given the high level of resistance observed in this study, it is advisable that the choice of antimicrobials for treatment of RTIs be based on antibiograms. This will ensure use of the most efficacious antimicrobials and will minimize treatment failures among cases presented with RTIs.

## Background

Bacteria, including *Pastuerella multocida, Bordetella bronchiseptica*, Streptococci, Staphylococci, and *Pseudomonas* species have been reported in respiratory tract infections (RTIs) in dogs (1–3). Moreover, dogs with compromised immune systems, underlying clinical conditions or those that are unvaccinated are at a higher risk of RTIs compared to healthy dogs (1, 3–5). Clinical signs associated with RTIs in dogs are quite varied and include coughing, nasal irritation, nasal discharge, ocular discharge, fever, dyspnea, anorexia, pulmonary hemorrhage, pleural effusion, and necrotizing pneumonia (6–8).

Treatment of RTIs is often supportive and prescriptions of antimicrobial drugs are dependent on the clinical presentations of the cases (9). Antimicrobials such as doxycycline, chloramphenicol, enrofloxacin, and amoxicillin are reported to be effective in treatment of cases of RTIs while trimethoprim-sulfamethoxazole, cephalosporins, and ampicillin are considered to be less effective (10). In South Africa, bacterial RTIs have not received much attention including assessment of antimicrobial susceptibility profiles of bacteria associated with these infections. Therefore, the efficacy of different antimicrobials for treatment of these infections is unknown. In view of this, the present study investigated the occurrence of RTIs, factors significantly associated with these infections, and antimicrobial susceptibility profile of isolates from dogs presented with these infections at a referral veterinary teaching hospital in South Africa.

## Methods

### Ethical Statement

This study was approved by the Animal Ethics Committee of the University of Pretoria (Approval Number: V055-17). Owners of all animals treated at the referral Veterinary Teaching Hospital where the data were obtained, sign a document granting permission for samples collected for the purpose of diagnosis to also be used for research purposes. All study findings are reported in aggregated form and therefore no patient identifiable information is included to ensure anonymity and confidentiality of patients and their owners are maintained.

### Study Design and Data Source

This retrospective study used laboratory records to estimate the burden and identify predictors of antimicrobial resistance among dogs presented with lower respiratory tract infections (RTIs) at a referral veterinary teaching hospital in South Africa. Samples of all the dogs included in the study were processed at the diagnostic laboratory of the referral veterinary teaching hospital. Microbiological analysis was performed using the methods described by Quinn et al. (11).

The data obtained from the records of these dogs included culture and antimicrobial susceptibility test results. A total of 157 dogs presented with lower respiratory tract infections at the referral veterinary teaching hospital between January 2007 and December 2013 and were included in the study. A dog was considered to have lower RTI based on clinical history and physical examination at the time of presentation at the referral veterinary teaching hospital. Clinical signs included coughing, tachypnea, dyspnea, and nasal discharge. In addition, to confirm the presence of lower RTIs, thoracic radiographs were done for all the animals in this study.

### Antimicrobial Susceptibility Testing

The isolates were subjected to antimicrobial susceptibility testing against a panel of 15 drugs using the Kirby-Bauer disc diffusion method. The following classes of antimicrobials were included in the panel: 30 μg amikacin (AK), 30 μg doxycycline (DOX30), 5 μg enrofloxacin (ENR), 10 μg gentamicin (CN), 10 μg ampicillin (AM) 10 μg penicillin G (P), 25 μg trimethoprim-sulphamethoxazole (co-trimoxazole) (SXT), 30 μg chloramphenicol (C), 30 μg cephalothin (KF), 30 μg kanamycin (K), 2 μg clindamycin (MY), 100 μg lincospectin (lincomycin hydrochloride and spectinomycin sulfate) (LS100), 5 μg orbifloxacin (OBX5), 20/10 μg Synulox (amoxicillin/clavulanic acid) (AMC20/10), and 15 μg tylosin (TY) (Oxoid Ltd., Cambridge, UK).

The laboratory from where the data were obtained, follows the Clinical and Laboratory Standards Institute (CLSI) procedures for isolation and determination of the susceptibility profile of the isolates (12–16). Depending on the susceptibility profiles, the laboratory classifies the isolates as susceptible, intermediate, or resistant. However, for purpose of this study, isolates that had been classified as intermediate resistance were re-classified as resistant. Furthermore, multidrug resistance (MDR) was defined as resistance to at least one agent in three or more antimicrobial categories (17). Extensive drug resistance (XDR), on the other hand, was defined as resistance to all but two antimicrobial agents in each category tested while pan-drug (PDR) resistance was defined as resistance to all antimicrobial agents tested (17).

### Data Management

The following variables were extracted from paper records and entered into an electronic database: age (in months), sex, breed, organism, antimicrobial agents tested, and the date of specimen submission. The American Kennel Club (AKC) breed classification system was adapted to classify dogs included in the study into the following categories: working, sporting, herding, hound, toy, terrier, non-sporting, and mixed breeds. The dataset was assessed for any missing information and none were identified.

### Data Analysis

#### Descriptive Analysis

All statistical analyses were performed in Statistical Analysis Systems (SAS) (18). Crude and factor-specific proportions of cases confirmed to have bacterial lower RTIs and isolates that exhibited antimicrobial resistance by breed, season, year, sex, age category, and specimen type were computed. Shapiro-Wilks test was used to assess if continuous variables were normally distributed. Non-normally distributed continuous variables were summarized using medians and interquartile ranges. Age was categorized into <2 years and ≥2 years. Temporal trends in the proportions of samples that were positive for bacterial RTIs and isolates that exhibited antimicrobial drug resistance were assessed using Cochran–Armitage trend test.

#### Inferential Statistics

Respiratory tract infections (RTIs) variable was defined as YES/NO based on whether or not the submitted sample was positive to at least one pathogen that causes respiratory tract infections. Each dog had only one sample submission over the study period and therefore only one outcome for RTIs (YES/NO) and hence there were no repeated measures associated with the RTIs variable. Simple associations between bacterial lower RTIs (Yes/No) and the covariates age, sex, breed and year were assessed using either Chi-square test or Fisher's exact test in situations where more than 20% of the cells had expected frequencies <5. Statistical significance was assessed using a critical *p*-value of 0.05.

#### Predictors of RTIs and Antimicrobial Resistance

Since there were no repeated measures associated with the outcome RTIs (YES/NO), ordinary logistic regression model was fit to the data to investigate its predictors in two steps. In the first step, ordinary univariable logistic regression models were fit to assess the relationships between the logit of bacterial RTIs and each of the suspected predictor variables: specimen type, season, sex, age, breed, and year. The potential predictors that were significant at a liberal *p* ≤ 0.2 in the univariable models were considered for inclusion in the multivariable model. In the 2nd step, an ordinary multivariable logistic regression model was built using a backwards elimination approach screening all suspected predictors that had *p* ≤ 0.2 in the 1st step. Statistical significance was assessed using a critical *p*-value of 0.05 during the 2nd step. Hosmer-Lemeshow goodness-of-fit test was used to assess model fit.

Samples from some dogs had multiple isolates of respiratory pathogens and therefore the MDR variable (coded as YES/NO) had repeated measures. Therefore, a Generalized Estimating Equation (GEE) model was fit to the data to adjust for the repeated structure in the data while investigating predictors of MDR. Again, the 1st step in model building involved assessing univariable associations between the logit of MDR and the predictors under investigation (sex, season, breed. year, sex, age, and specimen type). Predictor variables that were significantly associated with MDR at a liberal level of significance of 0.2 were offered for assessment in the manual backwards multivariable GEE model specifying binomial error distribution and logit link. All predictor variables were specified as fixed effects while the identification of the dog was specified as the repeated measure since multiple isolates were identified from the same dogs. Significance of fixed effects were assessed at a critical *p*-value of 0.05.

## Results

### Characteristics of Dogs Tested

Overall, 157 dogs were tested for bacterial lower RTIs. However, some of the records did not have complete information for all the variables and therefore the number of records used in different computations will vary depending on the number of records with complete data. The majority of samples tested were bronchoalveolar lavage (41.0%; 64/156) and transtracheal aspirate (39.1%; 61/156) (Table 1). Most dogs (42.7%; 67/157) were tested during summer months, while few were tested in Autumn (16.6%; 26/157). Similar proportions of male (51.4%; 72/140) and female (48.6%; 68/140) dogs were also tested. Sporting breeds had the highest (23.7%; 33/139) proportion of samples tested, while herding breeds had the least (4.3%; 6/139) (Table 1). More dogs ≥2years (61.8%, 97/158) compared to <2years (38.2%, 60/157) were tested.

**Table 1:** Summary statistics and results of univariable logistic regression models used to assess potential predictors of lower respiratory tract infections among samples from dogs presented at a referral veterinary teaching hospital in South Africa, 2007-2013.

**Samples tested**	**Samples tested**				**Respiratory infections**				**Univariable association test results**^**a**^			
	***n*^**d**^**	**%**	**95% CI**^**b**^		***n*^**e**^**	**%**	**95% CI**^**b**^		**OR^**c**^**	**95% CI**^**b**^		***p*-value**
Specimen												0.6848
Bronchoalveolar lavage	64	41.0	28.5	53.5	38	59.4	47.2	70.5	1.32	0.65	2.69	
Lung	27	17.3	9.2	25.4	14	51.9	34.0	69.3	0.98	0.39	2.42	
Transtracheal aspirate	61	39.1	26.9	51.3	32	52.5	40.2	64.5	Ref	–	–	–
Thoraco-centesis	4	2.6	0.6	5.8	0	0	0.0	0.0				
Season												0.0752
Summer	67	42.7	29.9	55.5	44	65.7	53.7	75.9	2.61	1.03	6.59	
Spring	33	21.0	12.0	30.0	15	45.5	29.8	62.0	1.14	0.40	3.21	
Winter	31	19.8	11.1	28.5	14	45.2	29.2	62.2	1.12	0.39	3.21	
Autumn	26	16.6	8.6	24.6	11	42.3	25.6	61.1	Ref	–	–	–
Sex												0.5617
Male	72	51.4	37.4	65.4	34	47.2	36.1	58.6	0.82	0.42	1.59	
Female	68	48.6	35.0	62.2	35	51.5	39.8	63.0	Ref	–	–	–
Breed												0.4494
Sporting	33	23.7	14.2	33.2	18	54.5	38.0	70.2	1.20	0.21	6.84	
Working	28	20.1	11.3	28.9	12	42.9	26.5	60.9	0.75	0.13	4.39	
Hound	21	15.1	7.5	22.7	8	38.1	20.8	59.1	0.62	0.10	3.82	
Terrier	17	12.2	5.4	19.1	5	29.4	13.3	53.1	0.42	0.06	2.82	
Toy	16	11.5	4.9	18.1	10	62.5	38.6	81.5	1.43	0.22	9.26	
Crossbreed	10	7.2	2.0	12.4	7	70.0	39.7	89.2	2.33	0.29	18.97	
Non-sporting	8	5.7	1.1	10.4	5	62.5	30.6	86.3	1.67	0.20	14.27	
Herding	6	4.3	0.3	8.4	3	50.0	18.8	81.2	Ref	–	–	–
Year	157	–	–	–	84	53.5	45.7	61.1		0.73	1.08	0.2392
Age												0.1080
<2years	60	38.2	31.0	46.0	37	61.7	49.0	72.9	1.711	0.889	3.296	
≥2years	97	61.8	54.0	69.0	47	48.5	38.8	58.3	Ref			

a Assessing association between respiratory infections and each of the variables listed in column 1. Note that none of the variables had significant association with log odds of respiratory infections at 5% level of significance. However, season had a potentially significant association with log odds of respiratory tract infections at 20% significance level.

b Confidence Interval.

c Odds Ratio.

d Number of samples tested.

e *Number of respiratory infections*.

### Respiratory Tract Infections

Of the dogs tested, 53.5% (84/157) were positive for bacterial RTIs. Bacterial RTIs were common in bronchoalveolar lavage (59.4%; 38/64) and transtracheal aspirate (52.5; 32/61) samples (Table 1). Summer had the highest proportion (65.7%; 44/67) of samples that were positive for bacterial RTIs. Equal proportion of samples from male (47.2%; 34/72) and female (51.5%; 35/68) dogs were positive for bacterial RTIs (Table 1). Simalrly, both dogs <2years (61.7%, 37/60) and ≥2years (48.5%, 47/97) tested positive for bacterial RTIs.

### Distribution of Bacterial Isolates

A total of 162 bacterial isolates were identified from the 84 bacterial positive samples. *Pasteurella* spp. were the most common (23.5%; 38/162) bacterial isolates followed by *Escherichia coli* (12.4%; 20/162), *Staphylococcus* spp. (9.3%; 15/162), *Streptococcus* spp. (8.6%;14/162), *Pseudomonas* spp. (5.6%; 9/162), *Enterococcus* spp. (4.3%; 7/162), *Lactobacillus* spp. (4.3%; 7/162), *Acinetobacter* spp. (3.7%; 6/162), *Klebsiella* species (3.7%; 6/162), *Moraxella* species (3.7%; 6/162), and *B. bronchiseptica* (3.1%; 5/162). Other isolates identified that had percentages less than 2% were aggregated into “All others” category which comprised 17.9% (29/162) of the isolates.

### Predictors of Bacterial Respiratory Infections

**Figure 1:**
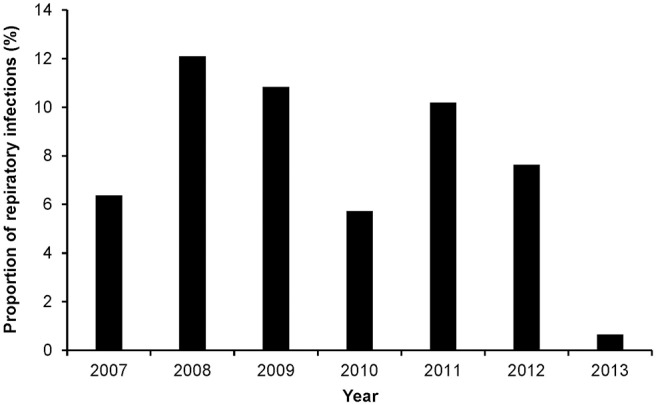
Annual proportions of respiratory tract infections among dog samples tested at a referral veterinary teaching hospital in South Africa, 2007-2013.

Season (*p* = 0.0752) and age (*p* = 0.1080) were the only factors that had a potential association with the log odds of RTIs at a relaxed alpha of 0.2 (Table 1). There was no significant (*p* = 0.2392) temporal trend in the annual proportions of respiratory tract infections between 2007 and 2013 (Figure 1, Table 1). Multivariable analysis revealed that there were no significant predictors of the log odds of bacterial lower RTIs at an alpha of 0.05 (Table 2).

**Table 2:** Adjusted associations for variables that had potential significant (alpha = 0.2) associations with log odds of respiratory infection.

**Variable**	**OR^**a**^**	**95% CI**^**b**^		***P*-value**
Age				0.1180
<2years	1.701	0.870	3.327	
≥2years	Ref	–	–	
Season				
Summer	2.624	1.030	6.684	0.0742
Spring	1.171	0.411	3.332	
Winter	1.124	0.389	3.244	
Autumn	Ref			

a Odds Ratio.

b Confidence Interval.

### Burden of Antimicrobial Resistance

Of the 162 isolates recovered, 153 were tested for antimicrobial susceptibility and 99.4% (152/153) of them were resistant to at least one antimicrobial drug while multidrug resistance was observed in 64.7% (99/153) of the isolates. Approximately a quarter (24.2%; 37/153) of the organisms isolated in this study were resistant to Beta-lactams.

**Figure 2:**
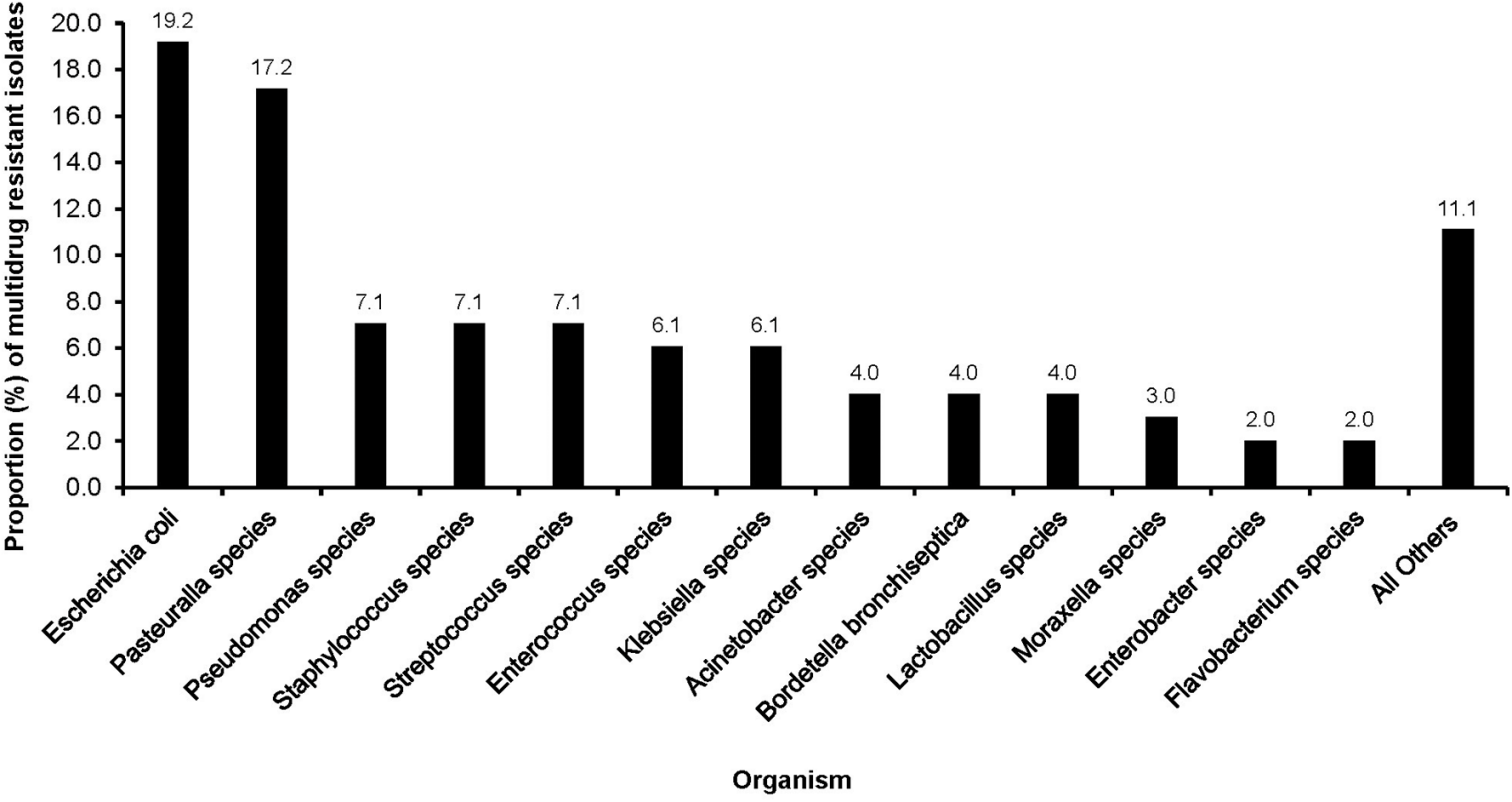
Distribution of multidrug resistant organisms isolated from clinical samples of dogs presented at a referral veterinary teaching hospital in South Africa, 2007-2013.

Multidrug resistance was common among *E. coli* (19.2%), *Pasteurella* spp. (17.2%) and *Pseudomonas* spp. (7.1%), *Staphylococcus* spp. (7.1%), and *Streptococcus* spp (7.1%). Low proportions of MDR were observed among *Moraxella* spp. (3.0%), *Enterobacter* spp. (2.0%), and *Flavobacterium* spp. (3.0%) (Figure 2).

**Figure 3:**
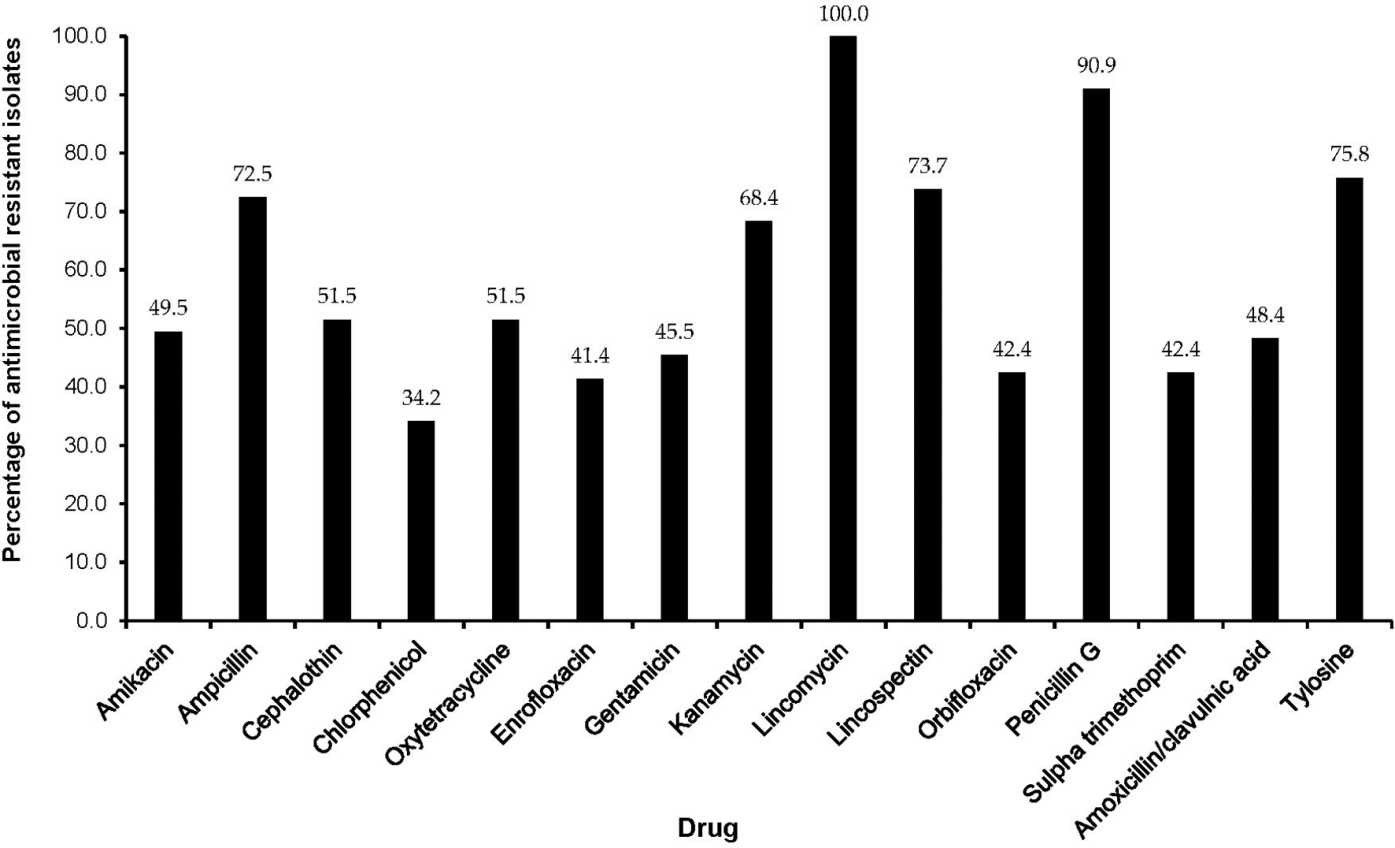
Antimicrobial resistance patterns of multidrug resistant isolates from respiratory infections of dogs presented to a referral veterinary teaching hospital in South Africa, 2007-2013.

**Figure 4:**
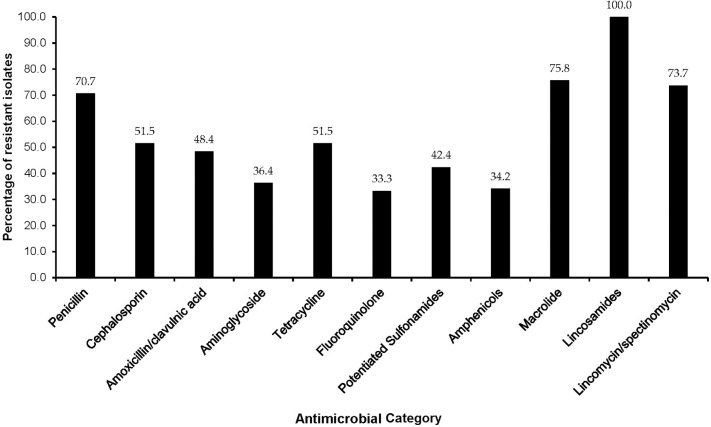
Distribution of multidrug resistant isolates by antimicrobial category among dogs presented at a referral veterinary teaching hospital in South Africa, 2007-2013.

The majority of MDR isolates were resistant to lincomycin (100%), penicillin-G (90.9%), tylosine (75.8%), lincospectin (73.7%), ampicillin (72.5%), and kanamycin (68.4%). There were low levels of resistance to chloramphenicol (34.2%) (Figure 3). With respect to antimicrobial categories, high levels of resistance among bacteria causing lower RTIs in dogs was observed to lincosamides (100%), penicillins (70.7%), macrolides (75.8%), and amoxicillin/clavulanic acid (48.4%) while fluoroquinolones (33.3%) isolates showed lower levels of resistance to (Figure 4).

### Predictors of MDR Among Isolates From Respiratory Tract Infections

Multidrug resistant isolates were common in both male (54.4%, 37/68) and female (68.3%; 41/60) dogs. Sporting breed had 68.8% (28.22/32) of MDR, working breed 60.9% (14/78) and toy breed 59.1% (13/22). Ninety percent (90.0%; 18/20) of MDR isolates were reported in the Autumn months, followed by Spring (66.7%; 16/24). Equal proportions of MDR isolates were reported in dogs <2years (62.9%, 44/70) and dogs ≥2years old (66.3%, 55/83).

**Figure 5:**
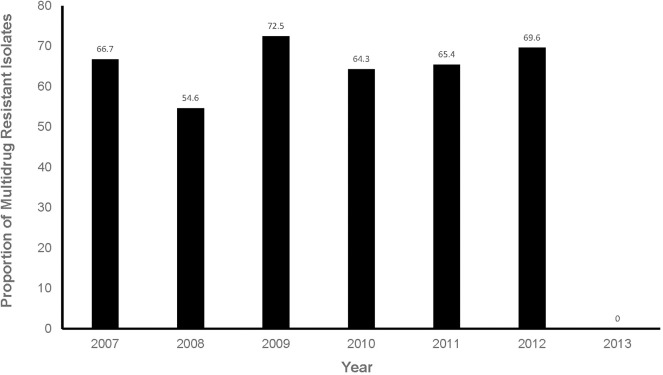
Proportions of multidrug resistant isolates from respiratory infection cases presented at a referral veterinary teaching laboratory in South Africa, 2007-2013.

Of the predictors investigated, sex (*p* = 0.1829) and season (*p* = 0.149) had a significant association with the logit of MDR isolates at a relaxed alpha of 0.2 (Table 3). There was no significant (*p* = 0.9165) annual temporal trend in the proportion of MDR isolates observed between 2007 and 2013 (Table 3, Figure 5). None of the variables had a significant association with MDR in the multivariable model at an alpha of 0.05 (Table 4).

**Table 3:** Summary Statistics and results of univariable logistic regression models used to assess potential predictors of multidrug resistant isolates from dogs present with lower respiratory tract infection at a referral veterinary teaching hospital in South Africa, 2007-2013.

**Variable**	**Resistant isolates**				**MDR**^**c**^ **isolates**				**Univariable association test results**^**a**^			
	***n*^**e**^**	**Percent**	**95% CI**^**b**^		***n*^**f**^**	**Percent**	**95% CI**^**b**^		**OR^**d**^**	**95% CI**^**b**^		***P*-value**
Sex												0.1829
Male	68	53.1	44.5	61.6	37	54.4	42.7	65.7	0.55	0.23	1.32	
Female	60	46.9	38.5	55.5	41	68.3	55.8	78.7	Ref	–	–	
Season												0.1249
Summer	86	56.2	48.3	63.8	51	59.3	48.7	69.1	0.16	0.03	0.77	
Spring	24	15.7	10.8	22.3	16	66.7	46.7	82.0	0.22	0.04	1.32	
Winter	23	15.0	10.2	21.6	14	60.9	40.8	77.8	0.17	0.03	1.01	
Autumn	20	13.1	8.6	19.3	18	90.0	69.9	97.2	Ref	–	–	
Breed												0.6931
Sporting	32	25.2	18.5	33.4	22	68.8	51.4	82.1	4.40	0.32	60.01	
Working	23	18.1	12.4	25.7	14	60.9	40.8	77.8	3.11	0.23	42.04	
Toy	22	17.3	11.7	24.8	13	59.1	38.7	76.7	2.89	0.21	39.45	
Hound	15	11.8	7.3	18.6	8	53.3	30.1	75.2	2.29	0.16	33.51	
Non-sporting	12	9.5	5.5	15.8	10	83.3	55.2	95.3	10.00	0.58	171.21	
Crossbreed	13	10.2	6.1	16.7	5	38.5	17.7	64.5	1.25	0.09	17.08	
Terrier	7	5.5	2.7	10.9	5	71.4	35.9	91.8	5.00	0.28	89.13	
Herding	3	2.4	0.8	6.7	1	33.3	6.2	79.2	Ref	–	–	
Age												
<2years	70	45.8	38.1	53.7	44	62.9	51.2	73.2	0.862	0.379	1.958	0.7243
≥2years	83	54.2	46.4	61.9	55	66.3	55.6	75.5				
Year												0.9165
2007-2013	152	99.4	96.4	99.9	99	64.7	56.9	71.8	1.013	0.790	1.300	

a Assessing association between multidrug resistance (MDR) and each of the variables listed in column 1. Note that none of the variables had significant association with log odds of MDR at 5% level of significance. However, sex and season had potentially significant associations with log odds of MDR at 20% significance level.

b Confidence Interval.

c Multidrug Resistance.

d Odds Ratio.

e Number of resistant isolates.

f *Number of multidrug resistant isolates*.

**Table 4:** Adjusted associations for variables that had potential significant (alpha = 0.2) associations with log odds of multidrug resistance (MDR).

**Variable**	**OR^**a**^**	**95% CI**^**b**^		***P*-value**
Sex				0.2033
Male	1.83	0.73	4.59	
Female	Ref	–	–	
Season				
Summer	1.25	0.37	4.2	0.3567
Spring	1.72	0.39	7.61	
Autumn	9.02	0.72	113.02	
Winter	Ref	–	–	

a Odds Ratio.

b *Confidence Interval*.

### Extensive Drug Resistance

Only 17.0% (26/153) of the organisms showed evidence of extensive drug resistance. Levels of extensive drug resistance was high among *Escherichia coli* (47.4%; 9/19) isolates, followed by *Pseudomonas* species (50.0%; 4/8) (Table 5). More female dogs (23.3%; 14/60) had extensively resistant organisms than male dogs (11.8%%; 8/68). The majority (20.8%, 5/24) of XDR isolates were reported in the spring (Table 5). XDR isolates were also reported in both dogs <2years (20.0%, 14/70) and those ≥2years old (14.5%, 12/83). Additionally, working (34.7%, 8/23), non-sporting (25.0%, 3/12) and sporting (21.9%; 7/32) breeds had the highest proportions of XDR isolates (Table 5). Very few of the isolates (3.3%; 5/153) were pan drug resistant.

**Table 5:** Distribution of extensively drug resistant (XDR) isolates from dogs with respiratory infections presented at a referral veterinary teaching hospital, 2007–2013.

**Variable**	**Resistant isolates**				**Extensively drug** **resistant (XDR) isolates**			
	***n*^**e**^**	**Percent**	**95% CI**^**b**^		***n*^**g**^**	**Percent**	**95% CI**^**b**^	
Sex								
Male	68	53.1	44.5	61.6	8	11.8	6.1	21.5
Female	60	46.9	38.5	55.5	14	23.3	14.4	35.4
Breed								
Working	23	18.1	12.4	25.7	8	34.7	16.4	57.2
Sporting	32	25.2	18.5	33.4	7	21.9	9.3	40.0
Non-sporting	12	9.5	5.5	15.8	3	25.0	5.5	57.2
Hound	15	11.8	7.3	18.6	2	13.3	1.7	40.5
Terrier	7	5.5	2.7	10.9	1	14.3	0.4	57.9
Toy	22	17.3	11.7	24.8	1	4.5	0.1	22.8
Crossbreed	13	10.2	6.1	16.7	0	0	0	24.7
Season								
Summer	86	56.2	48.3	63.8	16	18.6	11.8	28.1
Spring	24	15.7	10.8	22.3	5	20.8	9.2	40.5
Autumn	20	13.1	8.6	19.3	3	15.0	5.2	36.0
Winter	23	15.0	10.2	21.6	2	8.7	2.4	26.8
Age								
<2years	70	45.8	38.1	53.7	14	20.0	12.3	30.8
≥2years	83	54.2	46.4	61.9	12	14.5	8.5	23.6
Organism								
*Escherichia coli*	19	12.4	8.1	18.6	9.0	47.4	27.3	68.3
species								
*Pseudomonas* species	8	5.2	2.7	10.0	4.0	50.0	21.5	78.5
*Acinetobacter* species	6	3.9	1.8	8.3	2.0	33.3	9.7	70.0
*Klebsiella* species	5	3.3	1.4	7.4	2.0	40.0	11.8	76.9
All Others	115	75.2	67.8	81.3	9.0	7.8	4.2	14.2

b Confidence interval.

e Number of resistant isolates.

g *Number of multidrug resistant isolates*.

## Discussion

In this study we investigated the proportion of bacterial infections and antimicrobial resistance of isolates from dogs presented with suspected respiratory tract infections at a referral veterinary teaching hospital. In addition, we investigated factors significantly associated with bacterial respiratory tract infections and multidrug resistance among these cases. Respiratory tract infections among dogs presented at the veterinary hospital in South Africa was higher than the 20% reported by Daodu et al. (19) in Nigeria, 44% reported by Angus et al. (20), and 47.7% by Lavan and Knesl (3) both in the US. The observed difference between our findings and those of the other authors could be due to vaccination coverage against RTIs being lower in our study than in the other studies. In addition, the presence of underlying clinical conditions have also been reported to increase the risk of bacterial respiratory infections which were not assessed in this study due to unavailability of information (6). Since the veterinary teaching hospital is the only one in the country, it is also possible that cases treated at the hospital might be more complicated than those treated at other veterinary clinics and hospitals. Moreover, the hospital serves the as a referral hospital for veterinary clinics and hospitals north of Pretoria, Gauteng (21).

Based on the findings of this study, *Pasteurella* species was the predominant organism associated with bacterial respiratory tract infections. This is contrary to findings of a study done in the US among shelter dogs that reported that *Mycoplasma cynos* was the most common pathogen associated with RTIs (3). Our findings are also contrary to those of studies done in Japan (2) and the UK (22), that reported that *B. bronchiseptica* was the most frequently isolated bacteria in dogs with RTIs. In view of this, it appears that there is a variation in pathogens associated with RTIs based on the target population and geographic area.

It is worth pointing out that the veterinary teaching hospital cares mainly for referral patients that may have been treated at primary care clinics and hospitals. Therefore, the estimates obtained in this study may not be generalizable to a wider population of dogs because dogs with lower respiratory infections may be under- represented since only complicated cases potentially get sent to the referral hospital by the primary care providers. Moreover, since the dogs presented at the referral hospital may have been exposed to antimicrobial agents before being admitted at the referral hospital, they may have higher levels of resistance than the general population. Of concern from a clinical point of view is the observation of high levels of multidrug resistant isolates and the presence of XDR and PDR isolates. This is because the occurrence of multitdrug resistance among the isolates complicates treatment and negatively affects patient care and recovery (23, 24). Furthermore, pathogenic organisms have the ability to transfer resistance genes among themselves and other bacterial species (25). Given that transmission of resistant organisms between humans and dogs have previously been reported, this has grievous public health implications (26, 27). In light of this, there is a need for studies to document the role played by dogs as reservoirs of multidrug resistant bacteria associated with respiratory tract infections.

Similar to findings from our study, Daodu et al. (19) in Nigeria reported low proportions of resistance to fluoroquinolones (3.3%) and aminoglycosides (13.3%) from bacteria isolated from dogs with RTIs. Schwarz et al. (28) in Germany also observed low levels of resistance to fluoroquinolones and aminoglycosides among *P. multocida* and *B. bronchiseptica*. These results are encouraging as these groups of drugs are recommended for treatment of patients with mild to severe clinical signs of bacterial RTIs (8, 20, 29, 30). We observed over 50% resistance to penicillins, lincosamides, cephalosporins, tetracyclines, and macrolides among respiratory tract infections. In view of this, the use of these drugs in treatment of canine respiratory infections is likely to result in treatment failures among dogs presented at the teaching hospital.

The use of secondary data has inherent limitations. For instance, information about previous use of antimicrobials before sample collection was not available in this study and this could have influenced the proportion of respiratory organisms isolated in this study. Moreover, we only assessed hospitalized cases of respiratory infections and isolates from these cases have been reported to have a higher risk of resistance (21). Finally, the teaching hospital deals with referral cases from clinics and hospitals, which may have been exposed to antimicrobial agents hence potentially higher levels of resistance. In addition, isolates exhibiting intermediate resistance were re-classified as resistant and this could have slightly biased the results of antimicrobial susceptibility toward higher proportion of resistance among bacterial isolates observed in this study. Nonetheless, the results of this study contribute toward a better understanding of antimicrobial resistance among dogs with RTIs at the veterinary teaching hospital in South Africa.

## Conclusions

*Pasteurella* species are common causes of RTIs among dogs presented at the referral veterinary teaching hospital in South Africa. However, these estimates should be interpreted with caution because referral hospital tend to care for mainly complicated cases. Therefore, these estimates may not be generalizable to the general population since non-complicated are seen by primary care providers and never show up in the referral hospital implying potential underestimate the burden of the problem. Thus, the estimates can only be inferred to the population of dogs seen at referral hospitals. The selection of antimicrobial therapy for use in these patients should be based on results from antimicrobial susceptibility tests. This is because of the high levels of MDR isolates observed in this study as well as the high proportions of isolates that were resistant to penicillins, lincosamides, cephalosporins, tetracyclines and macrolides, and the presence of XDR and PDR among isolates. These finding are important for guiding future larger studies and has implications for public health because of the potential for transfer of infections from companion animals to humans.

## Data Availability Statement

The datasets generated or analyzed during the current study are not publicly available because they belong to a third party (the bacteriology lab of the University Pretoria Veterinary Teaching Hospital) but are available from the lab on reasonable request. Requests to access these datasets should be directed to [Daniel Nenene Qekwana, nenene.qekwana@up.ac.za].

## Ethics Statement

Owners of all animals treated at the referral Veterinary Teaching Hospital at the University of Pretoria sign a document indicating that they agree to the fact that samples collected for the purpose of diagnosis might, in addition, be used for research purposes.

## Author Contributions

DQ was involved in study design and data management and performed all statistical analyses and interpretation as well as preparation of the manuscript draft. VN and JO was involved in study design and editing of the manuscript. AO was involved in study design, data analysis and interpretation as well as extensive editing of the manuscript. All authors listed have made a substantial, direct and intellectual contribution to the work, and approved it for publication.

## Conflict of Interest

The authors declare that the research was conducted in the absence of any commercial or financial relationships that could be construed as a potential conflict of interest.

## Abbreviations

AKC: American Kennel Club
AM: Ampicillin
C: Chloramphenicol
CI: Confidence Interval
CLSI: Clinical and Laboratory Standards Institute
CN: Gentamicin
DOX: Doxycycline
ENR: Enrofloxacin
GEE: Generalized Estimating Equation
K: Kanamycin
KF: Cephalothin
MDR: Multidrug resistance
MY: Clindamycin
LS: Lincospectin
OB: Orbifloxacin
P: Penicillin
PDR: Pan-drug resistance
RTI: Respiratory tract infection
SXT: Trimethoprim-sulphamethoxazole
TY: Tylosin
UK: United Kingdom
US: United States
USA: United States of America
XDR: Extensive drug resistance.
